# The balance between NRF2/GSH antioxidant mediated pathway and DNA repair modulates cisplatin resistance in lung cancer cells

**DOI:** 10.1038/s41598-019-54065-6

**Published:** 2019-11-27

**Authors:** Matheus Molina Silva, Clarissa Ribeiro Reily Rocha, Gabriela Sarti Kinker, Alessandra Luiza Pelegrini, Carlos Frederico Martins Menck

**Affiliations:** 1 Department of Microbiology, Institute of Biomedical Sciences, University of São Paulo, São Paulo, Brazil; 2 Department of Experimental and Clinical Oncology, Federal University of São Paulo, São Paulo, Brazil; 3 Department of Physiology, Institute of Biosciences, University of São Paulo, São Paulo, Brazil

**Keywords:** Cancer therapeutic resistance, Non-small-cell lung cancer, DNA, Nucleotide excision repair, Prognostic markers

## Abstract

Lung cancer patients face a dismal prognosis mainly due to the low efficacy of current available treatments. Cisplatin is the first-line chemotherapy treatment for those patients, however, resistance to this drug is a common and yet not fully understood phenomenon. Aiming to shed new light into this puzzle, we used established normal and malignant lung cell lines displaying different sensitivity towards cisplatin treatment. We observed a negative correlation between cell viability and DNA damage induction upon cisplatin treatment. Interestingly, drug sensitivity in those cell lines was not due to either difference on DNA repair capacity, or in the amount of membrane ion channel commonly used for cisplatin uptake. Also, we noted that glutathione intracellular levels, and expression and activity of the transcription factor nuclear factor erythroid 2-related factor 2 (NRF2) were determinant for cisplatin cytotoxicity. Remarkably, analysis of gene expression in non-small cell lung cancer patients of the TCGA data bank revealed that there is a significant lower overall survival rate in the subset of patients bearing tumors with unbalanced levels of NRF2/KEAP1 and, as consequence, increased expression of NRF2 target genes. Thus, the results indicate that NRF2 and glutathione levels figure as important cisplatin resistance biomarkers in lung cancer.

## Introduction

Cancer is one of the main causes of morbidity and mortality worldwide, with a total annual economic costs of approximately US$ 1.16 trillion, and the number of cases are expected to rise 70% over the next two decades^1^. In 2018, it is estimated that different types of cancer will cause 610 thousand deaths in the United States alone, and the most common cause will be cancers of the lung, with 84 thousand deaths. Lung cancer can be divided in two main types – non-small-cell lung carcinoma (NSCLC) and small-cell-lung carcinoma (SCLC) – with the former accounting for 85% of the cases and being relatively insensitive to chemotherapy, and the latter being highly aggressive and invasive through the metastasis process. As a consequence, lung tumor patients face a very poor prognosis. For instance, in the U.S., only approximately 18% of patients diagnosed with lung cancer survive more than five years^2^.

Commonly, chemotherapy for lung cancer patients uses platinum-based compounds, especially cisplatin, which is also used for ovarian, testicle, and head and neck tumors^3^. Cisplatin is an alkylating agent that reacts with water when inside cells, replacing its chloride atoms with water molecules. This process results in highly reactive molecules that can covalently bind to DNA to form cisplatin-DNA adducts – including monoadducts, intrastrand crosslinks and interstrand crosslinks (ICL). ICLs are the most toxic DNA lesions caused by cisplatin, due to severe distortions in the DNA double helix that blocks replication and transcription, and, as a consequence, inducing cell death^4^. Another mechanism that has been described as responsible for cisplatin cytotoxicity is the induction of reactive oxygen species (ROS), compounds that interact with macromolecular components, such as lipids, proteins and DNA, generating lesions^5^.

Several mechanisms have been reported as responsible for cisplatin resistance, such as reduced drug uptake by a decrease in CTR1 expression, increased efflux by ABC transporters, augmented induction of stress response chaperones, induction of autophagy, inactivation of apoptosis pathway signaling, inactivation by thiol-containing proteins, and functional changes in DNA repair pathways^6–11^.

Since cisplatin cytotoxicity is mainly due to its ability to cause DNA damage, DNA repair capacity is expected to be one of most important, and therefore studied cisplatin-resistance mechanisms. The most relevant DNA repair pathways responsible for repair cisplatin-induced DNA damage are nucleotide excision repair (NER) and homologous recombination (HR). Importantly, ERCC1 (Excision Repair Cross-Complementing group 1) and XPF (Xeroderma Pigmentosum complementation group F) are essential proteins for both the NER and HR pathway. ERCC1 forms a heterodimer with the endonuclease XPF, and this protein complex is responsible for the incision of the DNA strand processing the damage. Not surprisingly, several reports described an association between ERCC1/XPF increased expression and activity with resistance to platinum compounds in many types of tumors^12^. The mismatch repair pathway (MMR) is also involved in cisplatin resistance, mainly as it recognizes post-replicative G/T mismatches induced by cisplatin adducts. MMR removes the base opposite to the adduct, which may cause a new mismatch, starting the pathway again and leading to the so-called futile cycle, generating double stranded breaks. Moreover, this process can inhibit the removal of cisplatin lesions by NER and ultimately leading to cell death. Thus, contrary to what is seen in NER, patients with tumors deficient in MMR show resistance to cisplatin^13^.

Other important resistance mechanisms prevent the drug from reaching the DNA and causing lesions. One of these mechanisms is the reduction in the intracellular accumulation of cisplatin due to an increase in the thiol-containing protein-mediated inactivation, such as glutathione (GSH), a highly abundant and low-molecular-weight tripeptide (Glu-Cys-Gly), well known antioxidant in cells. GSH can bind to and inactivate cisplatin through its highly reactive thiol group, preventing the drug from binding to DNA and cause damage^14,15^. The enzymes responsible for GSH synthesis, such as glutamate-cysteine ligase modifier subunit (GCLM) and the glutamate-cysteine catalytic subunit (GCLC), and enzymes related to GSH utilization, such as glutathione reductase, glutathione peroxidase and glutathione S transferase (GST), have their transcription regulated by the transcription factor NRF2 (nuclear factor, erythroid-derived 2-like 2 factor), known as the master regulator of antioxidant response^16^. Normally, NRF2 is attached to KEAP1 (Kelch-like ECH associated protein 1), promoting NRF2 proteasomal degradation. During oxidative stress situations, KEAP1 is oxidized and then NRF2 goes to the nucleus, promoting the transcription of many genes. The importance of NRF2 to cisplatin resistance has been demonstrated recently, and its overexpression has been correlated with higher resistance to several chemotherapeutic drugs in different types of cancer^17^.

The aryl hydrocarbon receptor (AhR) signaling pathway, activated by polycyclic aromatic hydrocarbons (PAHs) present in tobacco, is another important important mechanism, particularly in cigarette smoke induced lung cancer. This pathway is involved in xenobiotic metabolism, inducing the expression of several detoxification enzymes by binding in xenobiotic response elements (XRE) containing promoters^18^. There is a cross-talk between AhR and the NRF2 pathways, with NRF2 being capable of inducing AhR transcription, and also with the AhR pathway inducing NRF2 activation by ROS generation, highlighting the importance of NRF2 in lung cancer^19–21^.

In this work, the molecular mechanisms of resistance to cisplatin were investigated, focusing on DNA repair and NRF2/GSH pathways. Using either cisplatin sensitive or resistant established lung cancer cell lines, we showed that cisplatin induced cell death correlated with increased DNA damage induction, and although DNA repair shows important contributions to cellular resistance, it can not explain the cell viability differences among these cell lines. On the other hand, increased NRF2 induction and GSH levels were shown to correspond to increased tumor cell resistance to cisplatin. Thus, this work clearly indicates that NRF2/GSH pathway plays a primary role in cisplatin resistance in lung cancer cells.

## Results

### Increased cell death after cisplatin treatment is related to higher DNA damage induction

**Figure 1:**
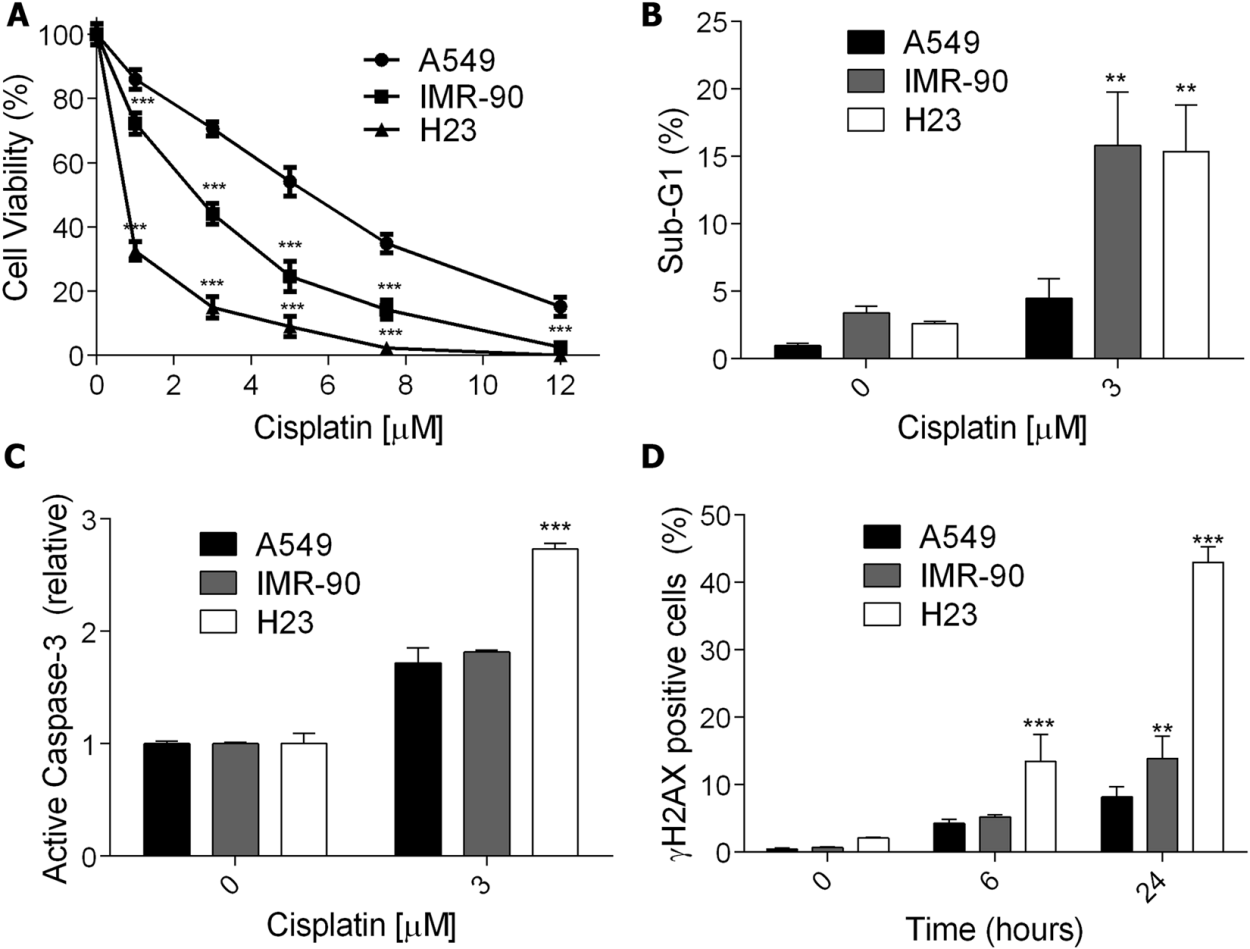
Cell death and DNA damage induction in normal and cancer lung cells after exposure to cisplatin. (**A**) A dose-response curve of three lung cell lines treated with increasing concentrations of cisplatin and analyzed after 72 h using the XTT assay. (**B**,**C**) The apoptotic fraction of lung cells treated with cisplatin for 72 h, analyzed as the sub-G1 population levels using flow cytometry of PI-stained nuclei or the fold increase of cells with active caspase-3 relative to control. **(D)** Flow cytometry analysis of γH2AX positive staining in lung cells upon treatment with cisplatin (5 μM) for 6 h and 24 h. Values are mean ± SEM of three independent experiments, *P < 0.05, **P < 0.01, ***P < 0.001.

The cell sensitivity to cisplatin treatment was evaluated in one normal lung fibroblast cell line (IMR-90) and two NSCLC cell lines (A549 and NCI H23). The cells were incubated with increasing doses of cisplatin, and after 72 h of treatment, the cellular viability was measured. As showed in Fig. 1A, A549 cells were more resistant to cisplatin treatment, whereas the NCI H23 cell line was the most sensitive, with IMR-90 cells showing an intermediary phenotype. In agreement, NCI H23 cells displayed higher levels of apoptosis induction when compared to A549 cells, as indicated by increased sub-G1 population (Fig. 1B), and caspase-3 activation (Fig. 1C).

DNA damage caused by cisplatin treatment can induce double-strand breaks (DSBs) on the DNA during its replication or repair process. In turn, DSBs lead to phosphorylation of the histone H2AX (γH2AX), which is widely used as a marker for genotoxic stress. Analysis of γH2AX-positive cells after cisplatin treatment showed that A549 cells had a low induction of DNA damage, while the NCI H23 lineage had a high induction at 6 h of treatment and displayed a threefold increase after 24 h. Again, the IMR-90 cell line showed an intermediary phenotype, with a significant increase in γH2AX staining only after 24 h (Fig. 1D, and Supplementary Fig. S1, where representative plots of flow cytometry are shown). *In situ* immunofluorescence for γH2AX was also performed for.cisplatin treated A549 and NCI H23 cells, with a clear increase of γH2AX foci in the damaged cells, particularly in NCI H23 cells (Supplementary Fig. S2). These data suggest that the increased resistance to cisplatin in tumors could be related to a lower induction of DNA damage.

### XPF silencing increases cisplatin induced cell death

**Figure 2:**
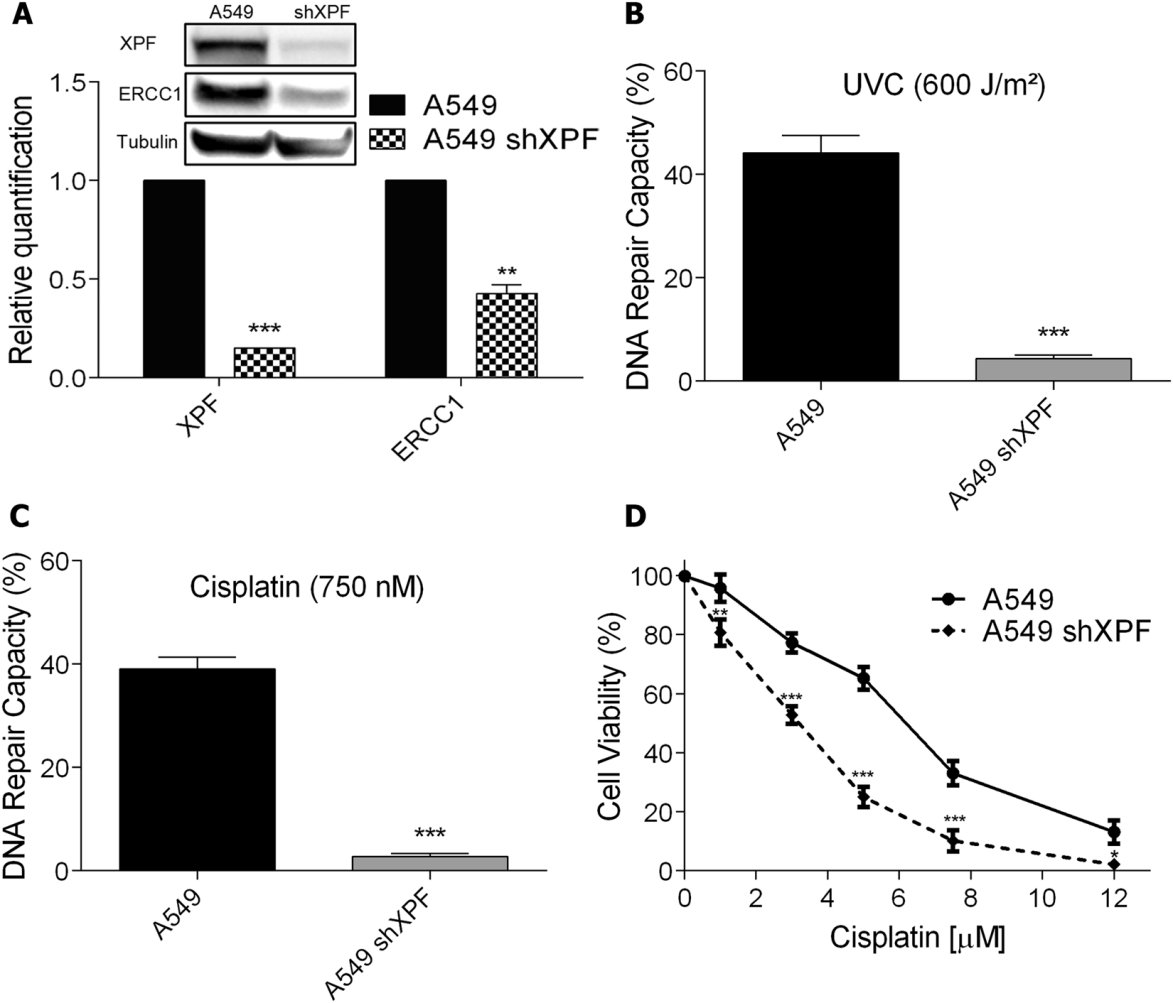
Knockdown of XPF and its effect on cell viability after exposure to cisplatin. **(A)** XPF and ERCC1 detection and relative quantification by western blot in A549 cells wild type or transduced with shXPF lentivirus. Full-lenght membranes are shown on Supplementary Fig. S6. **(B**,**C)** HCR assay with a luciferase plasmid irradiated with 600 J/m^2^ of UVC or treated with 750 nM of cisplatin, respectively. **(D)** A dose-response viability curve of A549 or A549 shXPF cell lines treated with increasing concentrations of cisplatin and analyzed after 72 h of treatment by XTT assay. Values are mean ± SEM of three independent experiments (two for the western blot experiments), *P < 0.05, **P < 0.01, ***P < 0.001.

Since a higher amount of DNA damage, as shown by the γH2AX analysis, correlated with increased cell death, we aimed to explore whether increased DNA repair capacity is responsible for A549 cisplatin resistance phenotype. Thus, NER endonuclease protein XPF was silenced in A549 cells (A549 shXPF) using shRNA lentiviral system. The silencing resulted in a substantial decrease in XPF protein levels, and, interestingly, also in the protein levels of its heterodimer partner ERCC1, suggesting that XPF is needed to maintain the stability of ERCC1 and prevent its degradation (Fig. 2A). These results are in agreement with observations that when XPF is not present, ERCC1 accumulates in the cytosol and does not translocate to the nucleus^22^. To gain further insights concerning the role of DNA repair as a resistance factor to cisplatin the host-cell reactivation (HCR) assay was performed. In this assay a damaged plasmid expressing a fluorescent protein reporter gene is transfected into the cells and the recovery of fluorescence detected by flow cytometry. The levels of fluorescence are directly affected by the DNA repair capacity of the cells. HCR analysis showed that A549 shXPF cells lose their capacity to remove UV (Fig. 2B) and cisplatin induced lesions (Fig. 2C). Notably, XPF-silenced cells displayed greater sensitivity to cisplatin treatment, similar to the cell viability observed for the normal cell line, IMR-90, as shown by the XTT cell viability assay and caspase-3 activation (Fig. 2D and Supplementary Fig. S3).

### DNA repair alone is not sufficient to determine cisplatin resistance in lung cancer cell lines

**Figure 3:**
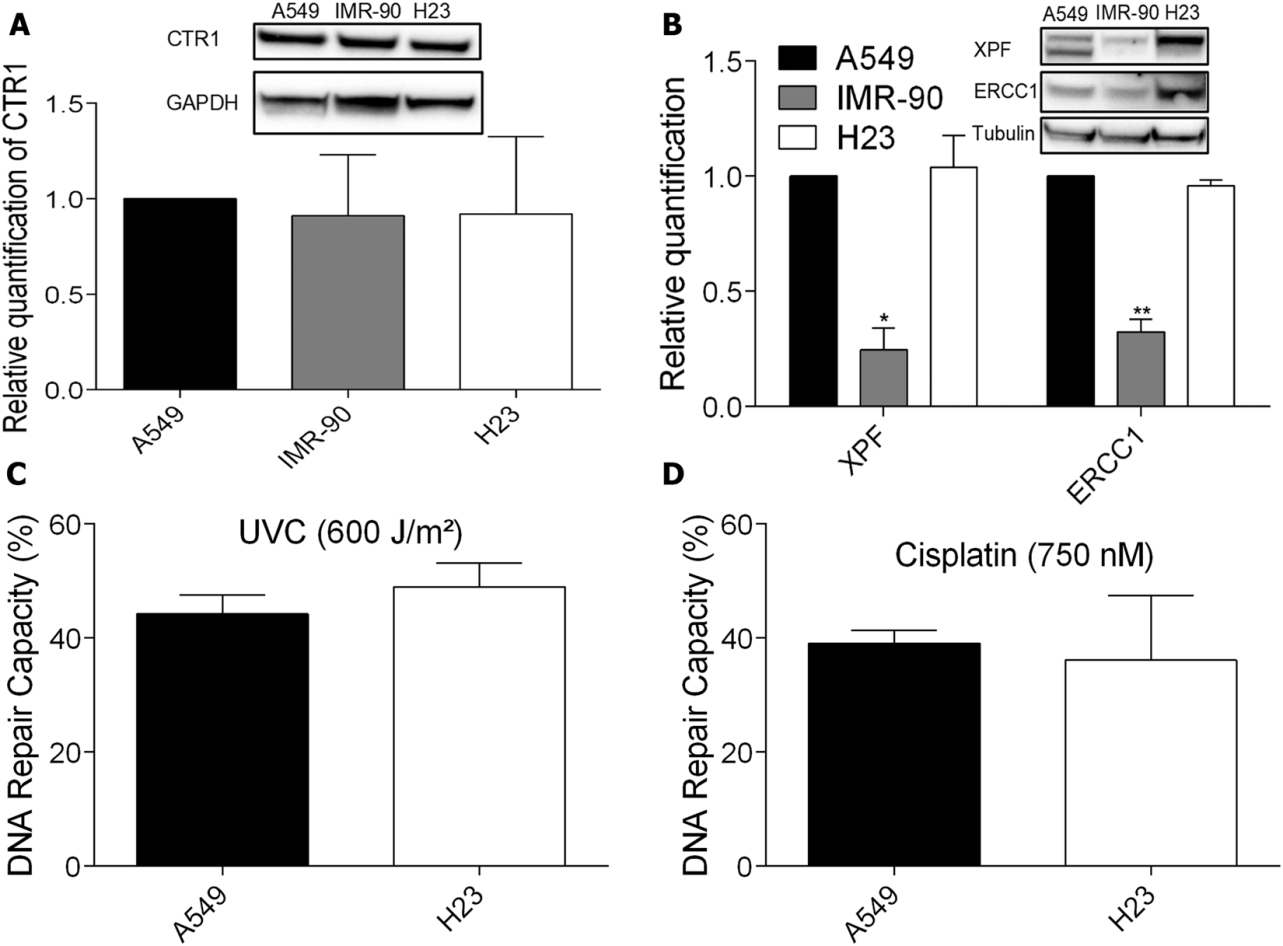
CTR1 status and DNA repair capacity in normal and cancer lung cells. (**A**,**B)** Human lung cancer whole-cell lysates analysis of CTR1, XPF and ERCC1 protein levels, respectively, by western blot. Full-lenght membranes are shown on Supplementary Fig. S6. **(C**,**D)** HCR assay of lung cancer cell lines transfected with a luciferase plasmid irradiated with 600 J/m^2^ of UVC or treated with 750 nM of cisplatin, respectively. Values are mean ± SEM of three independent experiments (two for the western blot experiments), *P < 0.05, **P < 0.01, ***P < 0.001.

One mechanism that could be responsible for the differential amount of DNA damage among the cell lines is cisplatin intracellular accumulation. Cooper transport channel (CTR1) is one of main mechanisms involved cisplatin cellular uptake. It has been observed that lower CTR1 expression leads to a decreased accumulation of intracellular cisplatin decreasing the amount of DNA lesions and conferring resistance to treatment^6^. As noticed on Fig. 3A, protein expression levels detected by western blot showed that there are no difference in the amount of the CTR1 protein among the three cell lines investigated, and therefore the DNA damage amount and sensitivity differences among them can not be explained by differential intracellular cisplatin accumulation.

With the possibility of each cell line receiving different amounts of the drug being discarded, we focused in understanding if the DNA repair capacity, as showed by the XPF knockdown in the most resistant lineage, could explain the different sensitivity to cisplatin among them. To address that, the protein levels of the heterodimer partners XPF and ERCC1 (Fig. 3B) were investigated. IMR-90 cell line displayed a decreased expression of both proteins, with levels similar to A549 shXPF cells, which could explain its reduced cell viability. However, surprisingly, the NCI H23 cell line, the most sensitive to cisplatin, showed the highest expression levels of these DNA repair proteins. To understand if, despite the high levels of XPF and ERCC1 proteins in these cells, the DNA repair capacity was reduced due to mutations in these or another proteins of the NER pathway, rendering it nonfunctional, the HCR assay was performed. Notably, it was observed that there are no differences between the repair capacity of both UV and cisplatin induced lesions in A549 and NCI H23 cells, the most and least resistant cells, respectively (Fig. 3C,D). Therefore, these results indicate that, although DNA repair is an important mechanism, other mechanisms may play a primary role in determining resistance to cisplatin, especially in NCI H23 cell line.

### NRF2/glutathione-mediated antioxidant defense pathway influences cisplatin resistance

**Figure 4:**
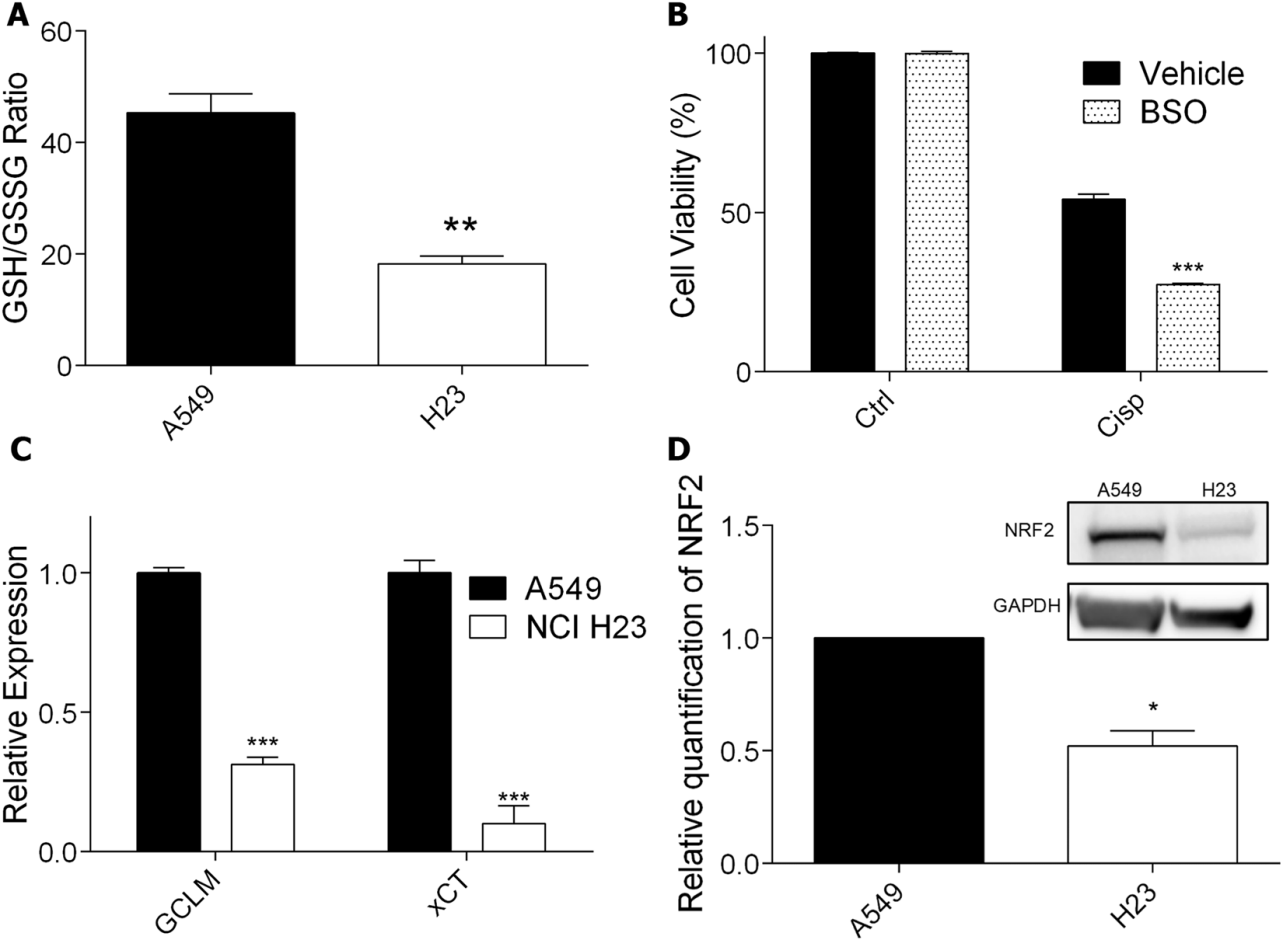
Glutathione production profile and NRF2 expression in lung cancer cells and effects in resistance to cisplatin. (**A)** Quantification of basal intracellular GSH/GSSG ratio in the lung cancer cell lines. **(B)** Cellular viability, as determined by XTT assay, in A549 cells treated with cisplatin (5 μM) and BSO (500 μM) for 72 h. **(C)** Quantification of GCLM and xCT mRNAs expression in lung cancer cells at basal levels, by real time PCR, normalized by GAPDH expression. **(D)** NRF2 protein level detection and relative quantification by western blot in lung cancer cells. Full-lenght membranes are shown on Supplementary Fig. S7. Values are mean ± SEM of three independent experiments (two for the western blot experiments), *P < 0.05, **P < 0.01, ***P < 0.001.

Another factor that could explain the different sensitivity between A549 and NCI H23 cell lines is an increased cisplatin detoxification, which can reduce cisplatin DNA damage, and as consequence, lower drug cytotoxicity. It is well established that one of the main factors that promote intracellular cisplatin detoxification is GSH, since it can bind covalently to the drug and prevent it to reach the DNA. Indeed, quantification of the intracellular ratio between reduced and oxidized GSH, an index of the intracellular amount of useful GSH, showed that A549 exhibited a higher ratio, indicating higher levels of reduced GSH and therefore showing an inverse correlation with the amount of DNA damage induced by cisplatin in both cell lines (Fig. 4A). To confirm that GSH levels may modulate cisplatin sensitivity, the A549 cell line was incubated with BSO (buthionine sulfoximine) – a well-known inhibitor of y-glutamylcysteine synthetase, an essential enzyme for the synthesis of GSH – and then treated with cisplatin. BSO alone did not result in decreased cell viability, however, when used in combination with cisplatin, it sensitized the cell line, resulting in a significant decrease in cell viability when compared to cisplatin alone (Fig. 4B and Supplementary Fig. S3).

Aiming to investigate the source of the difference in GSH levels between both lung cancer cell lines, the expression of GCLM (an enzyme responsible for GSH synthesis) and xCT (a subunit of an antiporter that exports glutamate while importing cysteine for glutathione synthesis) was assessed through real time PCR (RT-PCR). The results show that the mRNA levels of both genes are significantly decreased in the NCI H23 cell line, which may explain the reduced GSH levels observed in this cell line (Fig. 4C). Importantly, the expression of these genes is controlled by the transcription factor NRF2, and, in fact, the level of this protein was highly reduced in the most sensitive cell line, NCI H23, explaining the reduced expression of genes involved in GSH synthesis (Fig. 4D). *In situ* immunofluorescence of NRF2 also indicated this protein is highly expressed and present in the nucleus of A549 cells, with lesser expression in the nucleus of H23 cells (representative results shown in Supplementary Fig. S4). Other classical NRF2 target genes mRNA expression were also investigated by RT-PCR: Heme Oxygenase 1 (HO1) and NAD(P)H Quinone Dehydrogenase 1 (NQO1), and enzymes related to glutathione utilization, such as the Glutathione Peroxidases 1, 2 and 3 (GPx1, GPx2 and GPx3), with all of them having reduced expression in the NCI H23 compared to A549 cells (Supplementary Fig. S5).

### NRF2 overexpression induces cisplatin resistance

**Figure 5:**
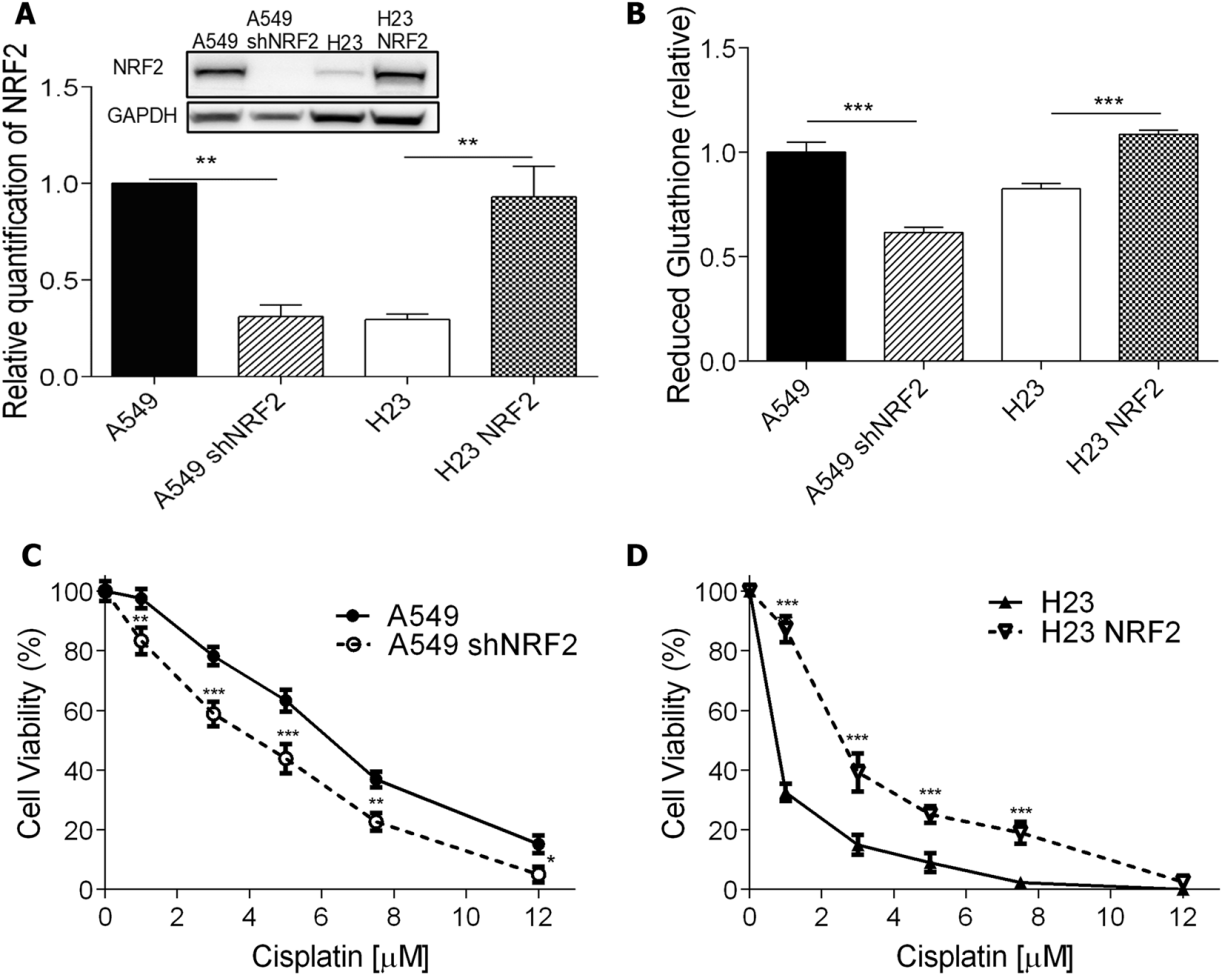
Cellular response of NRF2 silenced or overexpressed cells to cisplatin treatment. **(A)** NRF2 protein level detection and relative quantification by western blot in A549 cells parent or transduced with shNRF2 lentivirus recombinant vector (A549 shNRF2), and H23 cells parent or transduced with NRF2 overexpression lentivirus recombinant vector (H23 NRF2). Full-length membranes are shown on Supplementary Fig. S7. **(B)** Quantification of basal intracellular reduced glutathione in the different cell lines **(C**,**D)** Dose-response curves of A549 and A549 shNRF2 (**C**) and H23 and H23 NRF2 (**D**) cell lines treated with increasing concentrations of cisplatin and analyzed after 72 h of treatment by XTT assay. Values are mean ± SEM of three independent experiments (two for the western blot experiments), *P < 0.05, **P < 0.01, ***P < 0.001.

Based on these results, we hypothesized that levels of the transcription factor NRF2 may determine cisplatin sensitivity in lung cancer cell lines by regulating GSH production. In order to test this hypothesis A549 NRF2 knockdown (A549 shNRF2), and NCI H23 NRF2 overexpressing (H23 NRF2) cell lines were established. As shown in Fig. 5A, there was a substantial decrease in NRF2 protein levels in the A549 shNRF2 cell line, while the overexpression was capable of raising the amount of the transcription factor in H23 NRF2 cells to levels comparable to the A549 cells. Importantly, a significant reduction and increase on GSH reduced levels were observed in NRF2 knockdown and overexpression cell lines, respectively (Fig. 5B).

Notably, and confirming our hypothesis, A549 shNRF2 showed a higher sensitivity to cisplatin treatment, as shown by the XTT cell viability assay (Fig. 5C). In contrast, the overexpression of NRF2 in NCI H23 cells greatly induced resistance to cisplatin treatment, with almost 3-fold higher cell viability at low doses (Fig. 5D). Caspase activation was also performed and the data totally confirmed these observations, with higher sensitivity for A549 shNRF2 and increased resistant for H23 NRF2 cell lines (Supplementary Fig. S3).

### NRF2-KEAP1 expression balance changes the prognostic of lung cancer patients

**Figure 6:**
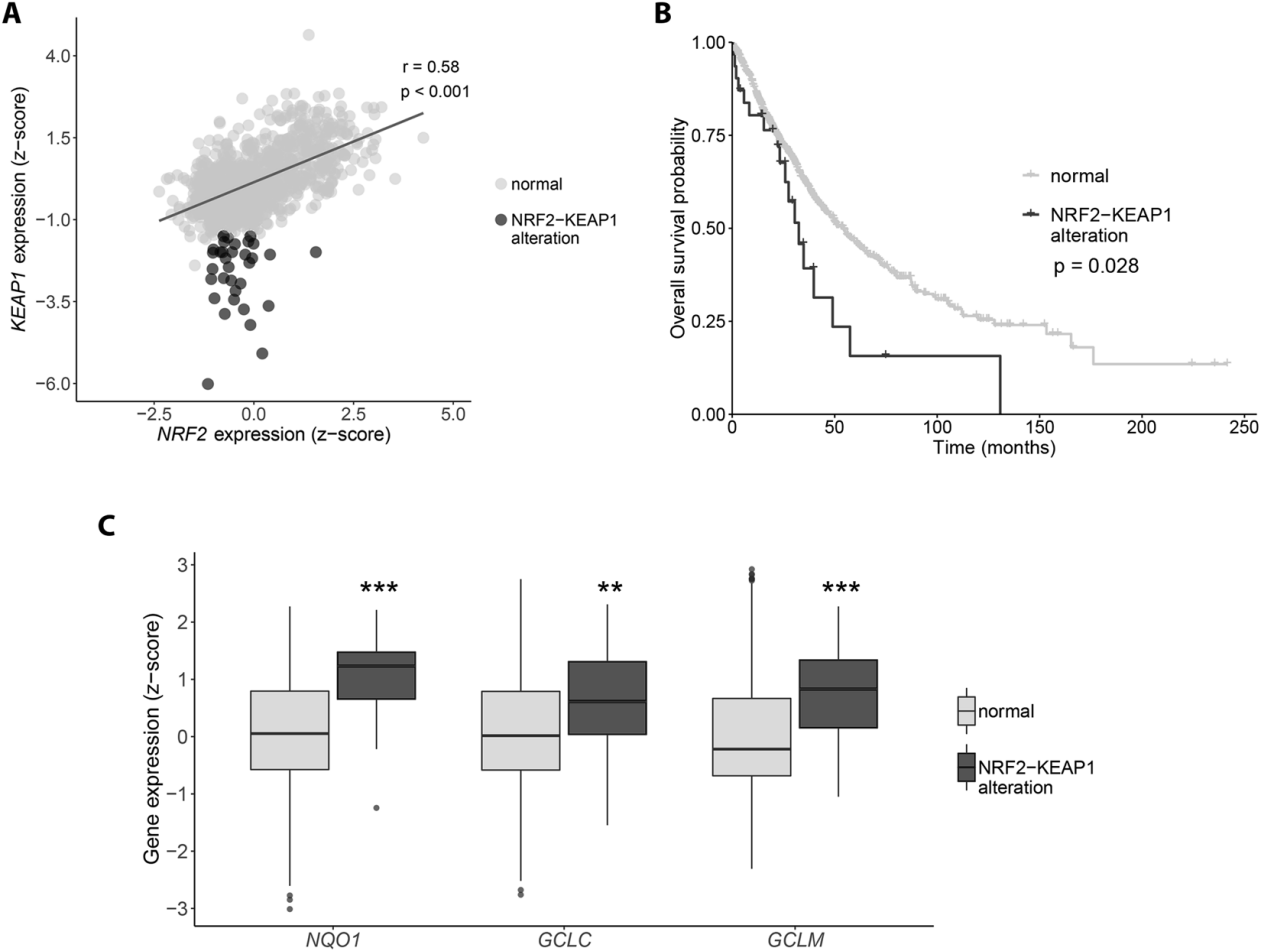
NRF2-KEAP1 expression balance impacts the overall survival in non-small cell lung carcinoma patients. (**A)** RNAseq analysis of NRF2 and KEAP1 expression in 1017 TCGA non-small cell lung carcinomas samples. Expression values were estimated using RSEM, upper quartile normalized, log2(x + 1) transformed and z-normalized. Expression correlation was calculated using the Pearson’s method. **(B)** Kaplan-Meier survival curves of patients divided according to the NRF2-KEAP1 expression balance. Comparisons were performed using the log-rank test. **(C)** Expression of NRF2 target genes in samples divided according to the NRF2-KEAP1 expression balance. The boxes extend from the 25th to the 75th percentile, the central bold line shows the median, and whiskers are drawn from minimum to maximum values within the 1.5 interquartile range. Comparisons were performed using the Student’s t-test. ***p < 0.001 and **p < 0.01 compared to the normal group.

Based on the evidence that NRF2 and GSH are determinants for cisplatin resistance in cell culture of lung cancer lines, we aimed to investigate if this is reflected in the prognostic of patients diagnosed with non-small cell lung cancer. For this purpose, we analyzed the gene expression data from TCGA for NRF2 and its inhibitor, KEAP1, observing that there is a strong positive correlation between them (Fig. 6A). However, there is a small subset of patients that does not obey this pattern, displaying median expression of NRF2 and low expression of KEAP1 − red dots (NRF2-KEAP1 alteration) − indicating a higher activity of the transcription factor in those tumors. Interestingly, the overall survival of this subset of patients compared with the remaining ones indicates that they have a significantly shorter survival rate (Fig. 6B). Importantly, in the patients bearing tumors with NRF2-KEAP1 alteration a significantly increase in expression of NRF2 target genes was observed, such as NQO1, and the enzymes responsible for GSH synthesis, GCLC and GCLM, which indicates that GSH levels are also higher (Fig. 6C). This data corroborates our *in vitro* analysis, since as we have shown, higher amount of NRF2 and GSH mediate tumor resistance to cisplatin treatment, ultimately leading to a worse prognosis for lung cancer patients.

## Discussion

Cisplatin, the main chemotherapeutic drug used to treat lung cancer, was the first FDA-approved platinum compound for cancer treatment, in 1978. It is clinically proven to combat different types of cancer, such as testicular, ovarian, cervical and head and neck, included on the World Health Organization’s list of essential medicines. Since its approval, 13 analogs have been evaluated in clinical trials, but only one, carboplatin, has achieved worldwide acceptance, due to its reduced side-effects, although being less effective, with the clinical standard dosage of carboplatin being a 4:1 ratio compared to cisplatin^23^.

DNA is considered the critical target for cisplatin’s cytotoxicity. Upon entering the cytoplasm, the chloride atoms on the drug are displaced by water molecule, resulting in a compound that binds to the N7 reactive center on purines and causes DNA damage. This process blocks cell division and results in apoptotic cell death^13^. Indeed, herein, the results show that lung cancer cell lines that are more sensitive to cisplatin treatment, as determined by XTT cell viability assays, presents higher levels of DNA damage, represented by increased phosphorylation of the H2AX histone, an important genotoxic stress marker^24^. Additionally, the DNA damage induction results in increased apoptosis, as indicated by a higher number of cells with degraded nuclei (sub-G1 population) and increased activation of Caspase-3.

However, the drug resistance of tumor cells limits the clinical use of cisplatin, which can be intrinsic or acquired. DNA repair activities of cancer cells clearly participate as a mechanism for cisplatin resistance. Particularly the heterodimer XPF-ERCC1, a rate-limiting factor for the NER pathway, that has single-strand DNA endonuclease activity and incises DNA on the 5’ side of cisplatin adducts, and also participate in the repair of double-strand breaks induced by ICLs^11^. Some studies show a correlation between the increase in the expression of these proteins and the repair of ICLs^25^. Cisplatin is highly effective in the treatment of testicular cancer, mainly due to the low levels of both proteins, and therefore ICL repair, in these tumors^26^. Also, patients with non-small cell lung carcinomas (NSCLC) with low levels of these proteins are more benefited by cisplatin chemotherapy. In melanomas and ovarian carcinomas, high levels of ERCC1 mRNA were observed after treatment with cisplatin^27^. Several inhibitors of XPF-ERCC1 have been shown to reduce NER activity and sensitize different types of cancer cells to cisplatin^13^. Furthermore, ERCC1 expression has been negatively correlated with survival after cisplatin chemotherapy in patients with non-small cell lung cancer (NSCLC) and is being exploited as a prognostic factor in the clinic^25^. In this study, a significant decrease in cell viability after XPF knockdown, which also reduces ERCC1 levels, was observed in the most resistant tumor cell line, confirming that DNA repair has a fundamental role in cisplatin resistance and variation in the protein levels of XPF and ERCC1 can explain, at least partially, the acquired resistance in tumors when compared to a normal fibroblast. Yet, this is not true for all cases, since the XPF/ERCC1 heterodimer levels were increased in the most sensitive cell line and no difference in DNA repair capacity was detected comparing the resistant and sensitive tumor cell lines.

Increased inactivation of cisplatin by thiol-containing proteins is an important mechanism for tumor resistance, as showed for cervical cancers, osteosarcoma and glioblastoma cells^4,14,15,28^. One of these nucleophilic species is glutathione, a tripeptide that is synthesized in the cytosol from glutamic acid, cysteine and glycine, that exists in thiol-reduced (GSH) and disulfide-oxidized (GSSG) forms, with the former representing around 98% of intracellular glutathione under physiological conditions^29^. GSH can bind to cisplatin through its reactive thiol group and prevent the drug from reacting with the DNA, thus, preventing damage induction. Indeed, the GSH/GSSG ratio is greatly reduced in NCI H23 cell line, explaining the higher DNA damage induction in these cells causing higher cell sensitivity to cisplatin. Furthermore, by using BSO − a chemical inhibitor of glutamate cysteine ligase (GCL), an enzyme responsible for glutathione synthesis − we depleted glutathione levels in A549 cells and observed significant increase in sensitivity to cisplatin, confirming the importance of glutathione in determining cisplatin resistance.

The intracellular glutathione pool is regulated by a series of enzymes involved in its synthesis, utilization and recycling, and high expression levels of these genes have been seen to promote cisplatin resistance in lung adenocarcinoma cell lines, with the glutamate cysteine ligase catalytic subunit (GCLC) expression being proposed as a potential predictor of treatment failure^30^. Importantly, the expression of these genes is controlled by the transcription factor NRF2, and several studies have shown that high amounts of NRF2 can induce cisplatin resistance in ovarian, bladder, and head and neck cancers^31–33^. Under physiological condition, NRF2 is constantly being targeted for proteasomal degradation by KEAP1, a process that is interrupted upon oxidation situations. In that sense, loss-of-function mutations in KEAP1 gene – leading to higher NRF2 activation – have been identified in several human adenocarcinoma, with lung cancer being the type where this gene is most mutated^34^. These mutations changed KEAP1 residues necessary for binding NRF2, reducing its affinity and promoting NRF2 nuclear accumulation. The A549 cell line, resistant to cisplatin treatment, shows a somatic mutation at the first Kelch domain of KEAP1, necessary for its interaction with NRF2, and also methylated promoter, both features that could explain the high levels of NRF2 protein in this cell line^35^. On the other hand, the NCI H23 cell line, sensitive to cisplatin, shows a mutation at the IVR (intervining-region) domain of KEAP1. This domain is necessary for the interaction with Cullin 3 (Cul3), a subunit of the E3 ligase complex, that promotes ubiquitination of NRF2^36^. We hypothesize that this mutation could promote a more stable interaction of KEAP1 with Cul3, increasing NRF2 degradation and explaining the lower levels in this cell line. In this work, both enzymes responsible for glutathione synthesis and NRF2 protein levels were highly reduced in a cisplatin sensitive cell line and, moreover, overexpression of this transcription factor induces an increase in GSH levels and resistance to the treatment. In agreement, knockdown of NRF2 in a cisplatin resistant cell line depleted GSH and sensitized the cells to cisplatin. Also, the analysis of non-small cell lung carcinomas in the TCGA data bank showed that, although there is a strong correlation between NRF2 and KEAP1 gene expression, there is a subset of patients with median expression of NRF2 and very low expression of KEAP1. As expected, these tumors have in fact increased expression of NRF2 target genes, including enzymes necessary for GSH synthesis. Interestingly, the patients with this NRF2/KEAP1 unbalanced expression show a worse prognosis when considering their overall survival.

Together, these results strongly indicate that the NRF2/GSH antioxidant defense pathway plays an important role in conferring cisplatin resistance in lung cancer cell lines. Although DNA repair is an important marker for cisplatin resistance, we have shown that neither repair proteins expression nor DNA repair capacity analysis alone can predict patient response to the treatment. On the other hand, analysis of NRF2 and GSH amounts showed to be a valid and potent parameter to presume the lung cancer sensitivity to cisplatin treatment. These observations are in line with the fact that tumors of the lung are highly mutated in KEAP1, the inhibitor of this pathway. Altogether, these findings support the evidence that NRF2 is a relevant prognostic factor for lung cancer patients, since its activity status can predict the treatment outcome. Finally, we propose the use of BSO plus cisplatin combinatory therapy as a potential alternative treatment regimen that may overcome cisplatin resistance in lung cancer patients.

## Materials and Methods

### Cell lines and culture conditions

Human lung carcinoma cell lines A549 and NCI H23, and human normal lung cell line IMR-90 were kindly provided by Prof. Mari Sogayar (University of São Paulo, Brazil). A549 and IMR-90 cell lines were cultivated in DMEM (Invitrogen, Life Technologies, Carlsbad, CA, USA), and NCI H23 cell line was grown in RPMI medium (Invitrogen), at 37 °C in a humidified, 5% CO_2_ atmosphere. The culture media were supplemented with 1% antibiotic-antimycotic and 10% FCS (fetal calf serum; Cultilab, Campinas, SP, Brazil). The A549 and NCI H23 cell lines have missense substitutions (p.G333C and p.Q193H, respectively) in the KEAP1 gene.

### Cell survival measurement

2 × 10^4^ cells were plated in a 12 multi-well plate and incubated with different doses of cisplatin (Sigma-Aldrich, St. Louis, MO, USA) for 72 h. In some experiments cells were pre-treated, for 16 h, with 500 μM BSO (Sigma-Aldrich, St. Louis, MO, USA). After that, cells were washed with phosphate-buffered saline (PBS) and incubated with XTT reagent kit as recommended by the manufacturer´s instructions (Roche, Basel, Switzerland).

### Flow cytometry for sub-G1, active caspase-3 and γH2AX analysis

1.5 × 10^5^ cells were plated in a 12 multi-well plate and incubated with 3 μM cisplatin for 6, 24 and 72 h. Supernatant and attached cells were collected, fixed with 1% formaldehyde and then with 70% ethanol. Afterwards, cells were blocked, permeabilized and incubated with a primary mouse monoclonal antibody to γH2AX (Ser-139) (Upstate Biotechnology, Lake Placid, NY, USA) diluted 1:500 for 16 h at 4 °C, followed by incubation with anti-mouse FITC secondary antibody (Sigma-Aldrich) diluted 1:200 for 2 h at room temperature, or with a mouse anti-active caspase 3 (BD, Pharmigen, San Diego, CA, USA) diluted 1:50 for 16 h at 4 °C. In both cases, cells were then stained with propidium iodide (PI) at room temperature for 1 h, in PBS containing 20 μg/mL PI (Sigma-Aldrich), 200 μg/mL RNase A and 0.1% Triton X-100.

### Western blot

Cells were lysed and the protein concentration was quantified using the Pierce BCA Protein Assay kit (Thermo Scientific, Rockford, IL, EUA). Proteins were separated by electrophoresis on a SDS-polyacrylamide gel and transferred to a nitrocellulose membrane (GE Healthcare, Waukesha, WI, USA). Membranes were blocked with 5% (w/v) milk powder in PBS for 1 h and incubated for 16 h at 4 °C with one of the following primary antibodies: rabbit anti-SLC31A1/CTR1 (Abcam, Cambridge, UK), mouse anti-GAPDH (Santa Cruz Biotechnology, Dallas, TX, USA), mouse anti-XPF (Thermo Fisher, Waltham, MA, USA), mouse anti-ERCC1 (Santa Cruz Biotechnology), mouse anti-Tubulin (Abcam), rabbit anti-NRF2 (Abcam). Afterwards, the membranes were incubated with the correspondent secondary antibody and a chemiluminescent HRP substrate (Merck Millipore. Burlington, MA, USA) was used to develop the membranes. Each blot was performed twice, and quantification of protein levels was done with the ImageJ software^37^.

### Host cell reactivation assay

The experiments of host cell reactivation assay were performed as previously described^38,39^. Briefly, pHIV-dTomato (Addgene #21374, Cambridge, MA, USA) plasmid was irradiated with 600 J/m^2^ of UVC light or incubated with 750 nM cisplatin for 3 h at 37 °C to induce DNA damage. In a 6 multi-well plate, 1.5 × 10^5^ cells were plated and transfected with 900 ng of pHIV-dTomato and 100 ng of pIRES2-EGFP (Clontech #6029-1, Mountain View, CA, USA), a transfection efficiency control, using Lipofectamine 3000 Transfection Reagent (Invitrogen, Life Technologies). One day after plasmid transfection, cells were collected and red and green fluorescence were measured by flow cytometry.

### Real-time PCR

RNA from cells was extracted using PureLink RNA Mini kit (Invitrogen) and cDNA was prepared using a High Capacity cDNA Reverse Transcription kit (Applied Biosystems, Life Technologies). Afterwards, 3 μL of diluted cDNA, 6 μL of SYBR green master mix, 0.5 μL of 10 mM reverse and forward primers and 2.5 μL of nuclease-free water were combined for each reaction to determine gene expression by quantitative PCR (Q-PCR), which was carried out using the 7500 Real-Time PCR System (Applied Biosystems). Relative expression of GCLM (Fwd: 5′-CCACCAGATTTGACTGCATTTG-3′/Rev: 5′-GCTTCTTGGAAACTTGCTTCAG-3′), GPx1 (Fwd: 5′-GACTACACCCAGATGAACGAG-3′/Rev: 5′-TCGAAGAGCATGAAGTTGGG-3′), GPx2 (Fwd: 5′-GCTTCCCTTGCAACCAATTTG-3′/Rev: 5′-TTCTGCCCATTCACCTCAC-3′), GPx3 (Fwd: 5′-CTGCTTTCCCTGCTCCTG-3′/Rev: 5′-GCTCCGTACTCGTAAATGGTG-3′), HO1 (Fwd: 5′-AACTTTCAGAAGGGCCAGGT-3′/Rev: 5′-GTAGACAGGGGCGAAGACTG-3′), NQO1 (Fwd: 5′-CAGCGGCTTTGAAGAAGAAAG-3′/Rev: 5′-GGTCCTTCAGTTTACCTGTGAT-3′) and xCT (Fwd: 5′-CCTGGCATTTGGACGCTACAT-3′/Rev: 5′-TCAGAATTGCTGTGAGCTTGCA-3′) was calculated using the relative standard curve method, and normalized against GAPDH (Fwd: 5′-ACCCACTCCTCCACCTTTGA-3′/Rev: 5′-CTGTTGCTGTAGCCAAATTCGT-3′) as the housekeeping gene.

### Glutathione quantification

Reduced and oxidized glutathione levels were quantified using the GSH/GSSG-Glo Assay (Promega, Madison, WI, USA), following the manufacturer’s instructions. Briefly, 1 × 10^4^ cells were plated in white, opaque, 96 multi-well plates and, after 24 h, they were lysed by using either a total glutathione lysis reagent or an oxidized glutathione lysis reagent. Then, luciferin generation reagent was added to the wells and the plate was incubated 30 min at room temperature, followed by addition of a luciferin detection solution and incubation of 15 min at room temperature. Luminescence was measured using a Glomax-Multi+ Luminometer (Promega), and serial dilutions of a GSH standard solution was used to generate a standard curve.

### The cancer genome atlas (TCGA) data analysis

TCGA gene expression and clinical data from 1017 non-small lung carcinomas were downloaded from the UCSC XENA Browser (http://xena.ucsc.edu) in July, 2018. Gene expression data were generated using the Illumina HiSeq. 2000 RNA sequencing platform, quantified using RSEM, upper quartile normalized, log_2_(x + 1) transformed, and z-normalized. Analysis of survival was performed using Kaplan-Meier curves and the log-rank test. We used the two-sided Student’s t-test to perform two-group comparisons and the Pearson’s method to calculate correlations.

### Statistical analysis

Data is presented as the mean plus standard error of the mean (SEM) of at least two independent experiments and statistical significance among data sets were accessed by applying two-way ANOVA followed by Bonferoni post-testing, always in comparison to the resistant cell line (A549), using Prism 6 software (GraphPad Software Inc., CA, USA). Differences were considered significant for p-values lower than 0.5.

## Supplementary information

The balance between NRF2/GSH antioxidant mediated pathway and DNA repair modulates cisplatin resistance in lung cancer cells

## Data Availability

The datasets generated during and/or analysed during the current study are available from the corresponding author on reasonable request.
