# Supplementary material for: The balance between NRF2/GSH antioxidant mediated pathway and DNA repair modulates cisplatin resistance in lung cancer cells
Source: Sci Rep. 2019 Nov 27;9:17639. doi: 10.1038/s41598-019-54065-6 (PMC6881285; doi:10.1038/s41598-019-54065-6)
Supplement: Supplementary file 1 — The balance between NRF2/GSH antioxidant mediated pathway and DNA repair modulates cisplatin resistance in lung cancer cells [file 41598_2019_54065_MOESM1_ESM.docx]

**The balance between NRF2/GSH antioxidant mediated pathway and DNA repair modulates cisplatin resistance in lung cancer cells**

SILVA MM, ROCHA CRR, KINKER GS, PELEGRINI AL, MENCK CFM

Authors’ full names:

Matheus Molina Silva¹, Clarissa Ribeiro Reily Rocha^2^, Gabriela Sarti Kinker^3^, Alessandra Luiza Pelegrini¹ and Carlos Frederico Martins Menck ¹^,*^

Affiliation:

¹ Department of Microbiology, Institute of Biomedical Sciences, University of São Paulo, São Paulo, Brazil; matheusmolina@usp.br (M.M.S.); aleepelegrini@gmail.com (A.L.P.)

^2^ Department of Experimental and Clinical Oncology, Federal University of São Paulo, Brazil; [clarissa.rocha@unifesp.br](mailto:clarissa.rocha@unifesp.br) (C.R.R.R)

^3^ Department of Physiology, Institute of Biosciences, University of São Paulo, São Paulo, Brazil; gabriela.kinker@hotmail.com (G.S.K.)

Corresponding Author:

^*^ Carlos Frederico Martins Menck, Department of Microbiology, Institute of Biomedical Sciences, University of São Paulo, Av. Prof. Lineu Prestes, 1374, São Paulo, SP 05508-900, Brazil. cfmmenck@usp.br (C.F.M.M.); Tel.: +55-11-3091-7499

**Supplementary Materials and Methods**

**Immunofluorescence:** Cells adhered in coverslips were washed three times with PBS and fixed with ice-cold methanol for 15 minutes. Coverslips were again washed three times with PBS and incubated with a mouse monoclonal antibody to γH2AX (Ser-139) (Upstate Biotechnology) or rabbit monoclonal antibody to phospho NRF2 (S40) (Abcam) diluted 1:100 or 1:50, respectively, in PGN-saponin (0.2 % gelatin, 0.1 % NaN3 and 0.25% saponin in PBS) for 16 h at 4 ºC. Coverslips were washed with PBS and incubated with Alexa Fluor 488 IgG anti-mouse or anti-rabbit (Life Technologies), diluted 1:100 in PGN-saponin for 1 h at room temperature. After washing with PBS, coverslips were prepared using Fluoroshield with DAPI (Sigma-Aldritch) and pictures were taken using the fluorescence microscope AxioVert.A1 a with 100X objective.

**Supplementary Figures**


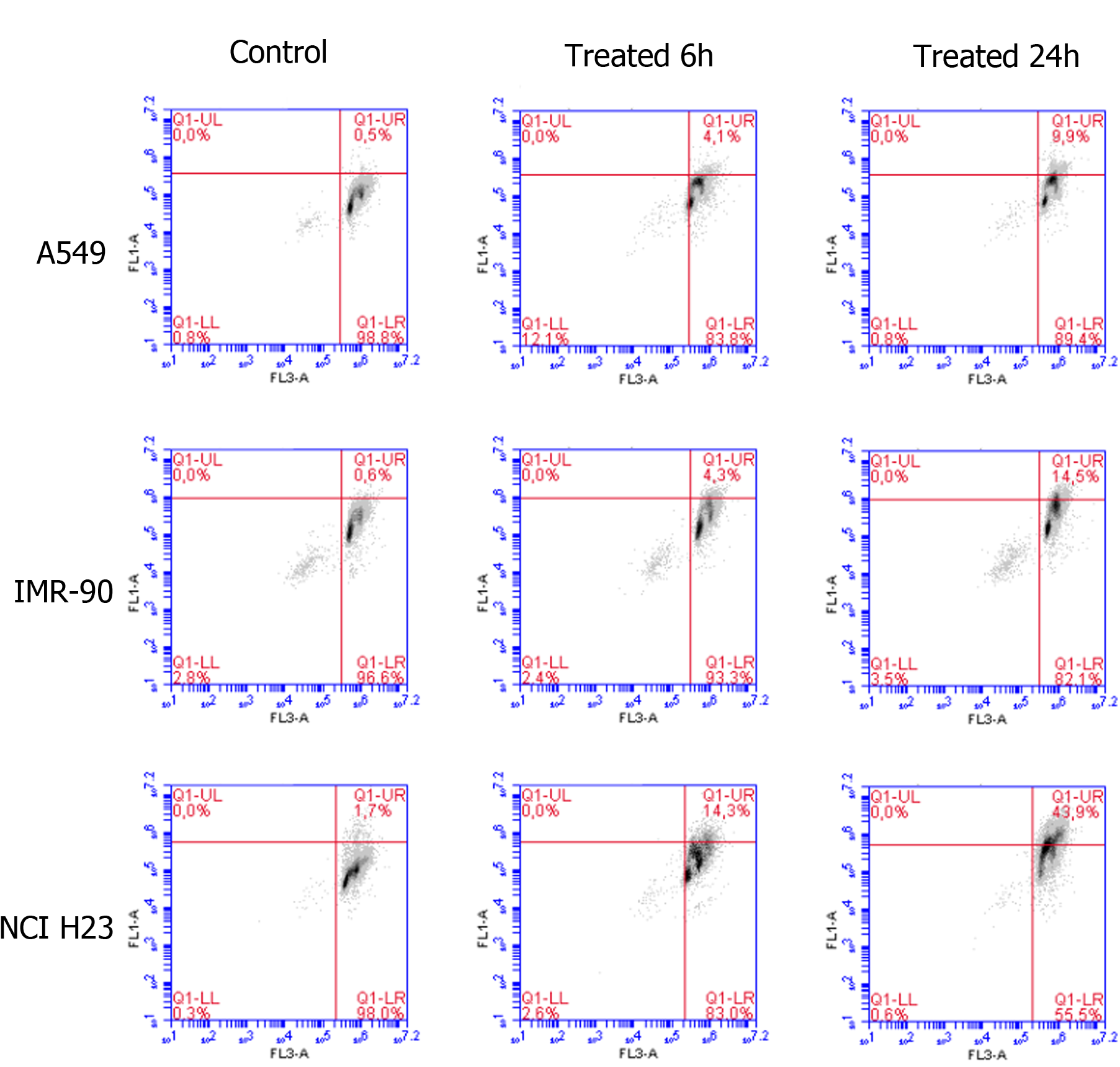
**Supplementary Figure S1 –** Representative plots of the flow cytometry traces for γH2AX analysis showed in Figure 1.


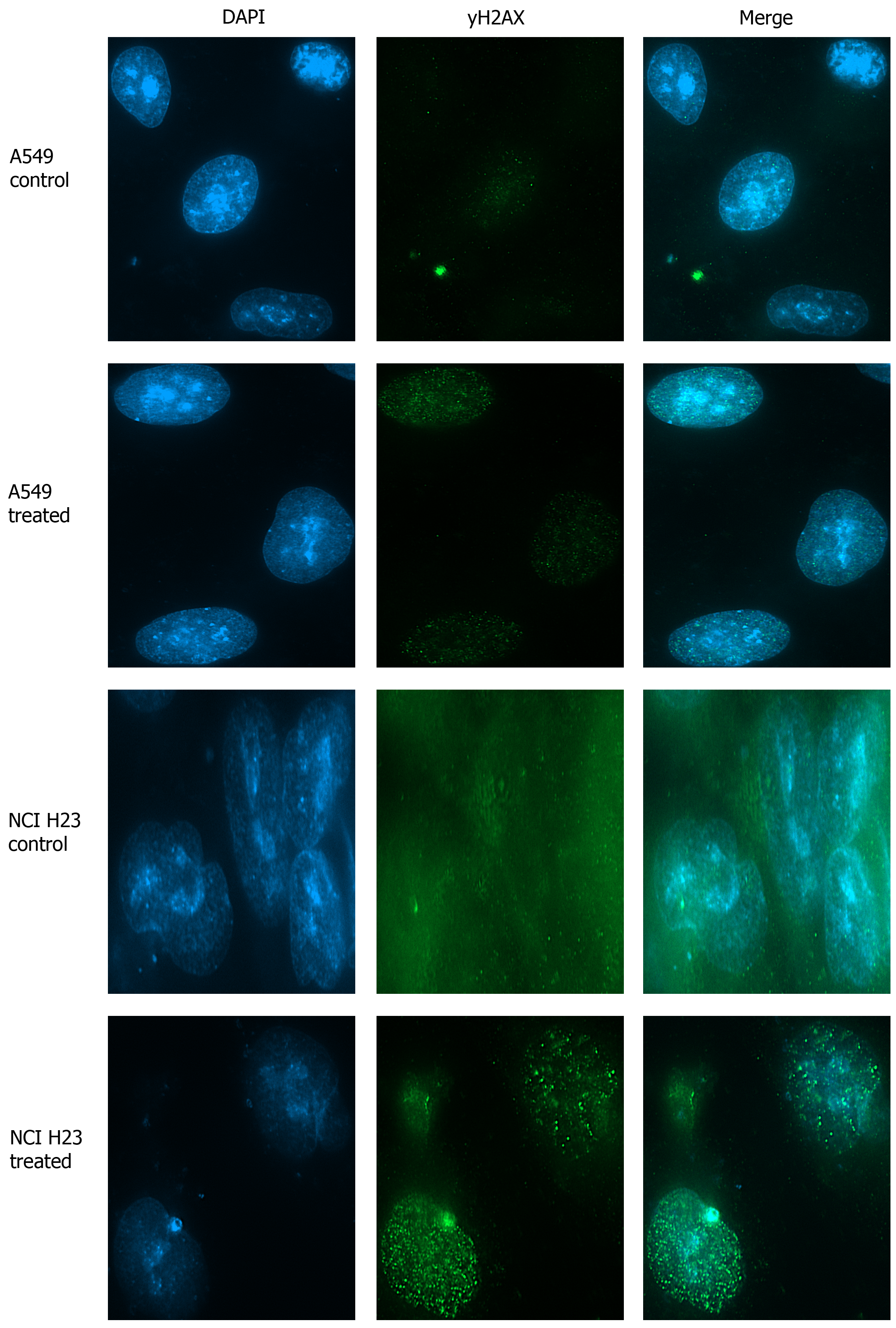
**Supplementary Figure S2 –** Immunofluorescence of γH2AX foci in the nucleus of A549 and NCI H23 cell lines after treatment with 5 μM of cisplatin for 24 hours.


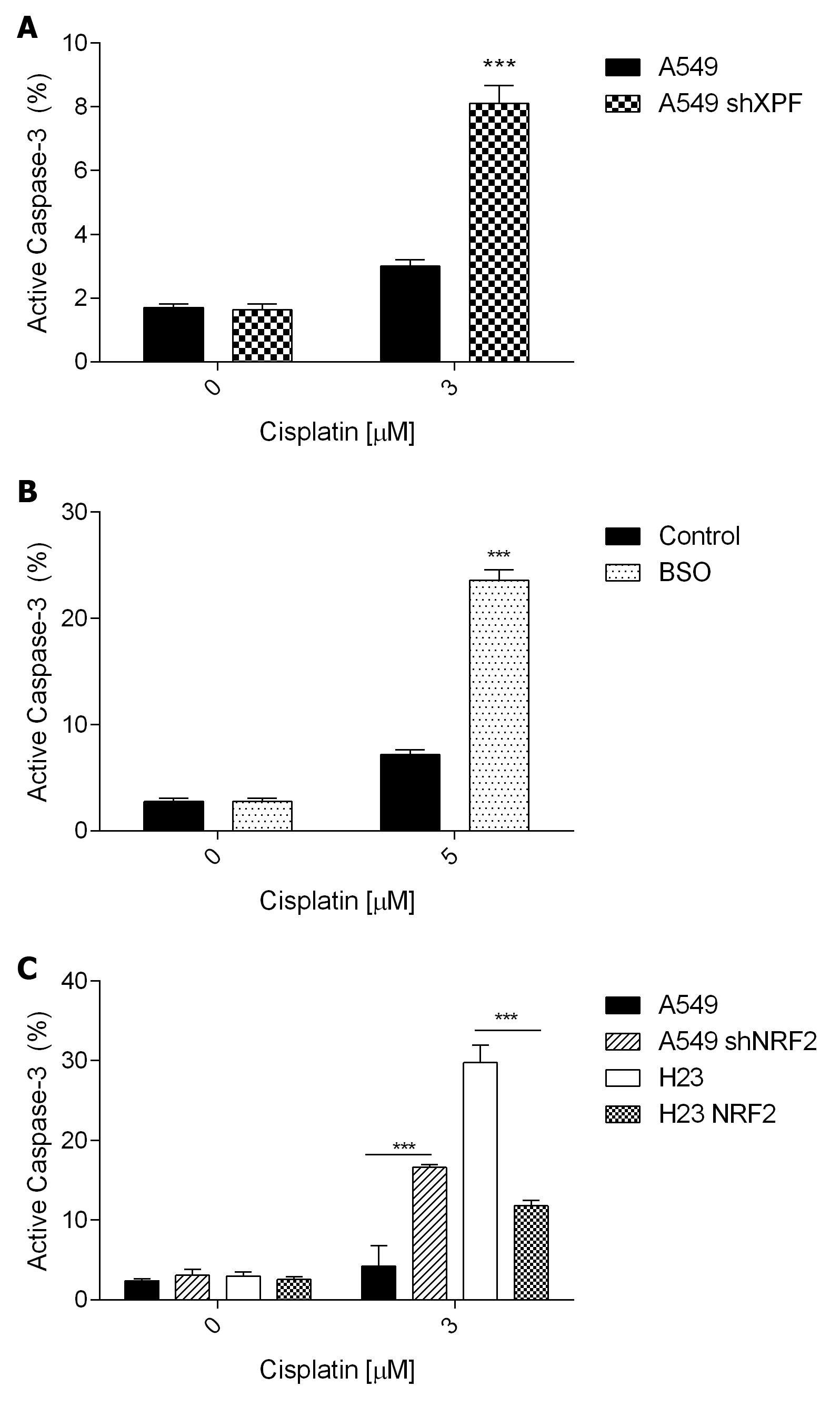
**Supplementary Figure S3 –** Active caspase-3 quantification by flow cytometry related to the XTT cell viability experiments presented in **(A)** Figure 2, **(B)** Figure 4 and **(C)** Figure 5. Values are mean ± SEM of three independent experiments, ***P<0.001.


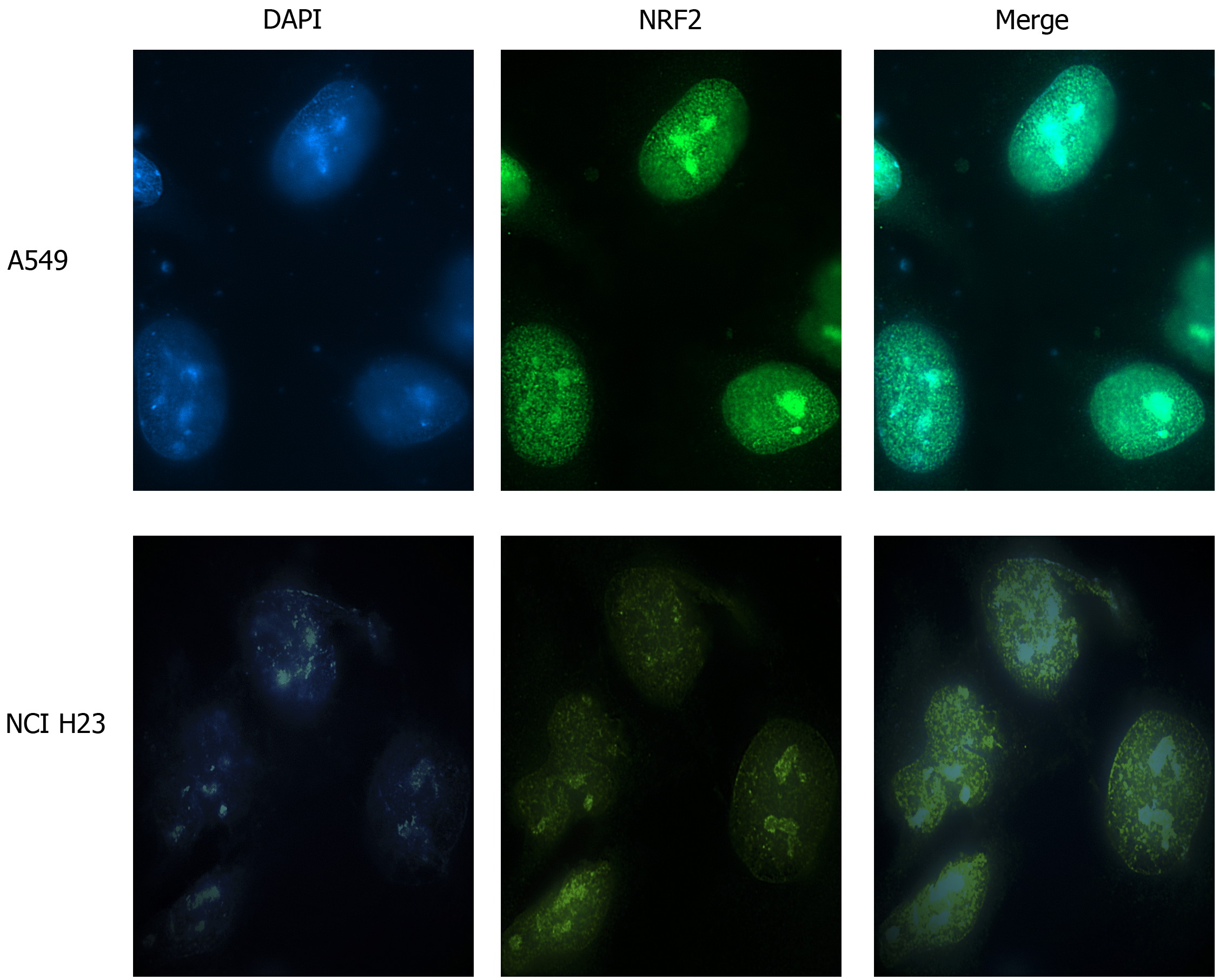
**Supplementary Figure S4** – Immunofluorescence of basal NRF2 levels in the nucleus of A549 and NCI H23 cell lines.



**Supplementary Figure S5 –** Quantification of HO1, GPx1, GPx2, GPx3 and NQO1 mRNAs (NRF2 targets) expression in lung cancer cells at basal levels, by real time PCR, normalized by GAPDH expression. Values are mean ± SEM of three independent experiments, ***P<0.001.


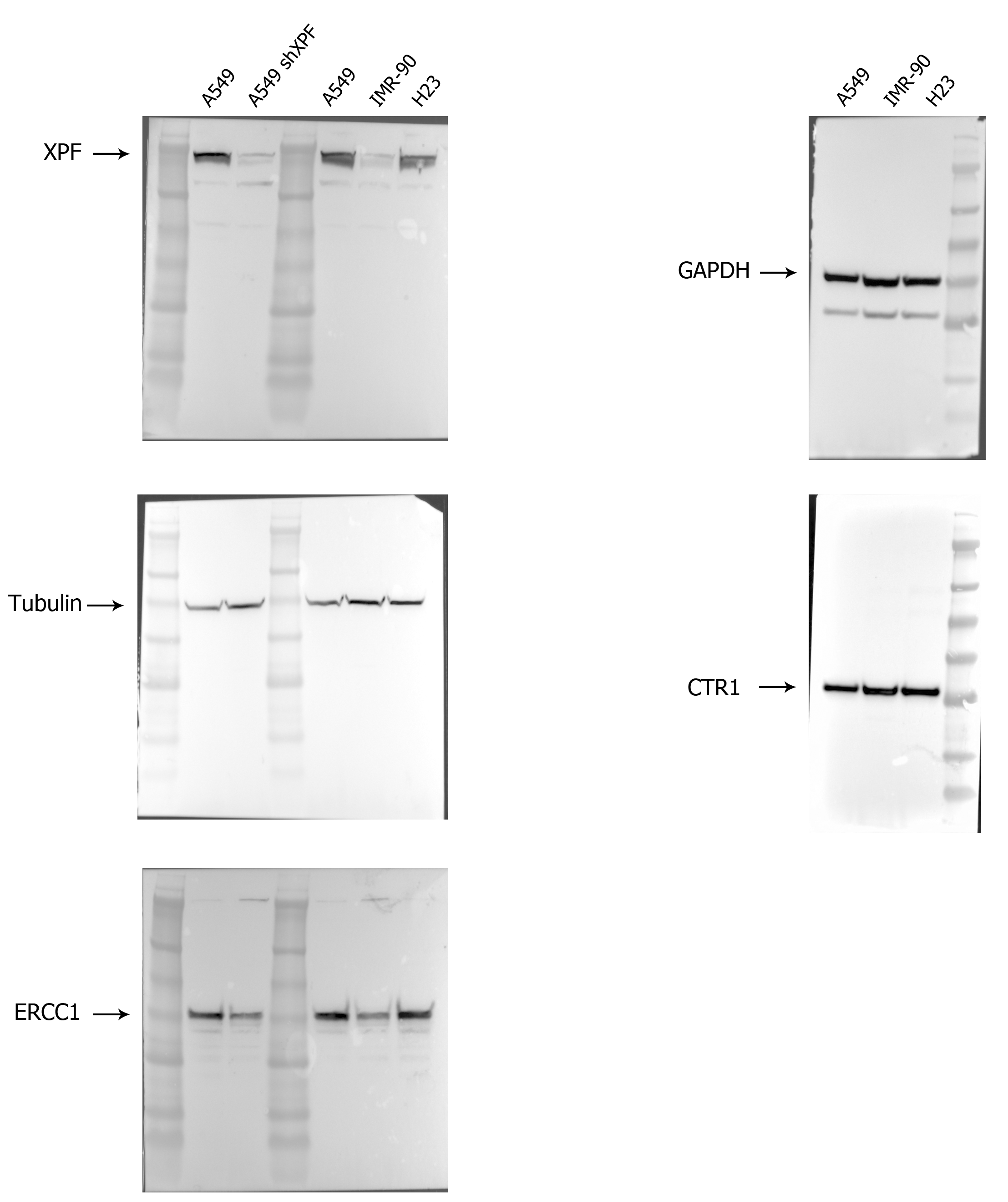
**Supplementary Figure S6 –** Representative images of full-size membranes for the western blots experiments displayed in Figures 2 and 3.


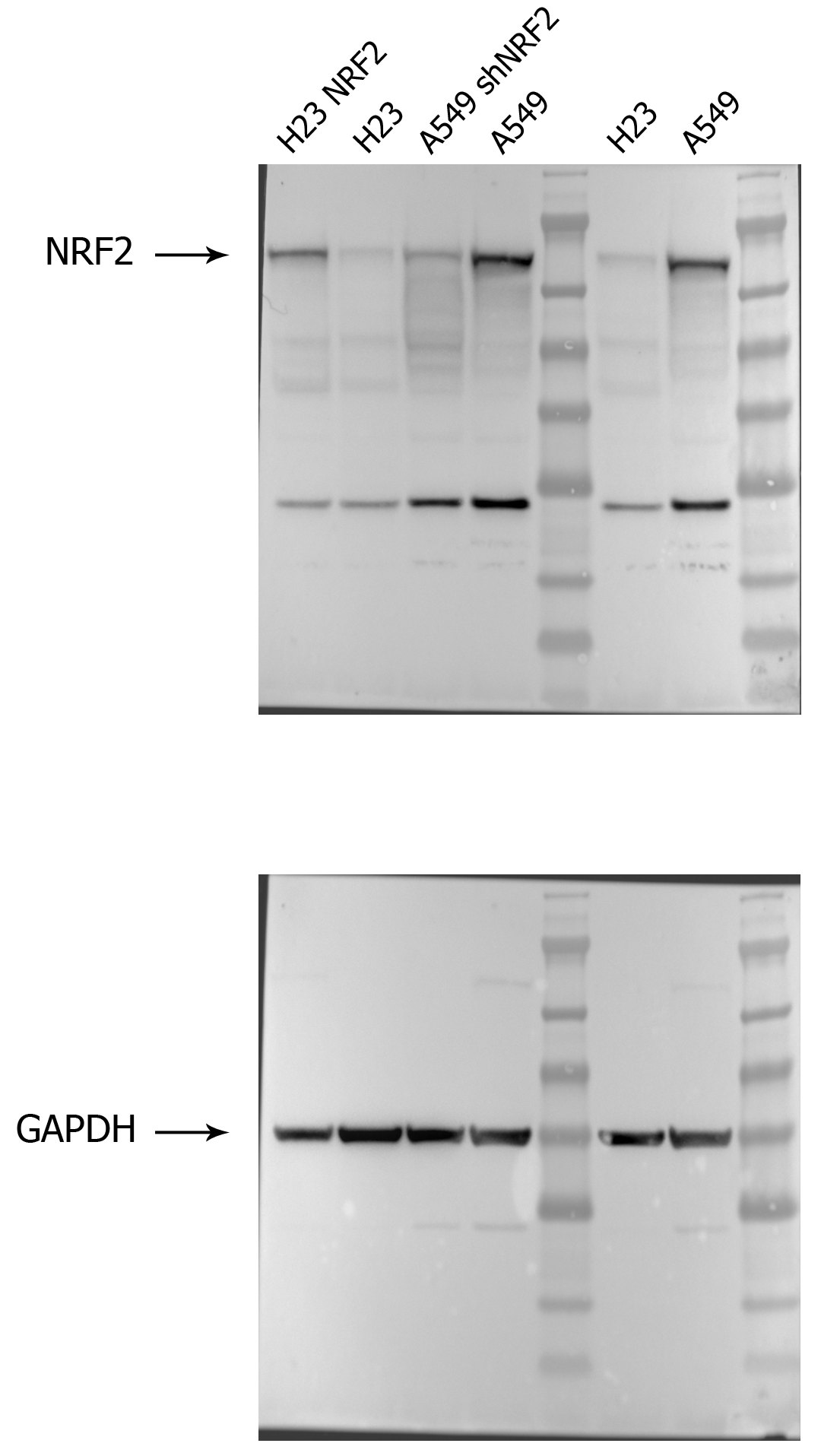
**Supplementary Figure S7** – Representative images of full-size membranes for the western blots experiments displayed in Figures 4 and 5.
